# Health care professional perspectives on vision screening in older adults who attend hospital following a fall: a focus group study

**DOI:** 10.1007/s41999-026-01486-y

**Published:** 2026-05-14

**Authors:** Aishah Baig, Kate Radford, Alison Cowley, Jignasa Mehta, Adam L. Gordon

**Affiliations:** 1 Queens Medical Centre, Nottingham University Hospitals NHS Trust, Derby Road, Nottingham, NG7 2UH UK; 2 Centre for Rehabilitation and Ageing Research, University of Nottingham, Medical School, Derby Road, Nottingham, NG7 2UH UK; 3 NIHR Nottingham Biomedical Research Centre, Queen’s Medical Centre, Nottingham University Hospitals NHS Trust, Derby Road, Nottingham, NG7 2UH UK; 4 East Midlands Regional Research Delivery Network, University Hospitals of Leicester NHS Trust, Paget House, 2 West Street, Leicester, LE1 6XP UK; 5 School of Allied Health Professions and Nursing, Institute of Population Health, University of Liverpool, Thompson Yates Building, Brownlow Hill, Liverpool, L69 3GB UK; 6 Wolfson Institute of Population Health, Queen Mary University of London, Charterhouse Square, London, EC1M 6BQ UK; 7 Academic Centre for Healthy Ageing, Barts Health NHS Trust, Whipps Cross Hospital, London, E11 1NR UK

**Keywords:** Falls, Vision screening, Vision, Aging

## Abstract

**Background:**

The assessment and management of impaired vision is included in falls prevention guidance. However, implementation is inconsistent. We conducted focus groups to explore the perspectives of Health Care Professionals (HCP) on vision screening in older adults attending acute hospitals following a fall.

**Methods:**

A focus group study was undertaken with HCPs from a single-acute hospital trust. Semi-structured topic guides were informed by the Consolidated Framework for Implementation Research (CFIR). Transcripts were first inductively then deductively coded using CFIR constructs. Demographic data were collected and summarized.

**Results:**

Five focus groups were conducted with 19 HCPs overall. Six interconnecting themes were identified, mapped to 14 CFIR constructs, relating to barriers and facilitators to vision screening. Barriers encompassed: lack of training, referral networks to manage impaired vision and prioritization of task-focused, rather than person-centered care, in the acute setting. Facilitators included: perceived mission alignment, adequate training, tools, guidance on roles, responsibilities and management pathways, integration of eye care professionals in multi-disciplinary falls care and time in job plans.

**Conclusions:**

HCPs were motivated to vision screen and felt it aligned with person-centered falls care, however there were individual-, structural-, and organization-level barriers related to staff capability and opportunity to implement vision screening in the acute setting. Multi-component and multi-level interventions and implementation strategies are needed to integrate eye care professionals into the falls multi-disciplinary team, engage supportive leaders, develop an effective vision screening assessment, define roles, responsibilities, and management pathways, and organize individual training and time allocation for staff to perform screening.

**Supplementary Information:**

The online version of this article (https://doi.org/10.1007/s41999-026-01486-y) contains supplementary material, which is available to authorized users.

## Key summary points

**Aim:**

This study aimed to explore barriers and facilitators to vision screening in older adults attending hospital following a fall, from the perspectives of the falls Multidisciplinary Team (MDT) in an acute hospital.

**Findings:**

Perceived capability, lack of referral pathways, integration of eye care professionals in falls MDTs, and undefined roles were barriers to vision screening. Vision screening aligned with perspectives of person-centered falls care but conflicted with task-focused acute care.

**Message:**

Multi-component and multi-level interventions and implementation strategies targeting individual-, structural-, and organizational-level barriers could facilitate vision screening for older adults attending hospital following .

**Supplementary Information:**

The online version of this article (https://doi.org/10.1007/s41999-026-01486-y) contains supplementary material, which is available to authorized users.

## Introduction

Globally, falls occur at least once a year in approximately a third of people aged ≥ 65 years and the prevalence increases with age. [1, 2] Falling can have serious physical [3–5] and psychosocial implications for affected individuals, [6–8] including increased mortality. [9] Worldwide, health and social care services face mounting costs related to falls management, [10–12] due to aging populations and the expected increase in number of falls [13, 14].

Impaired vision almost doubles the risk of falling [15] and is more prevalent in older adults acutely admitted to hospital with falls, compared to those admitted without falls [16–18] and non-fallers in the community. [18–20] People who fall and attend an Emergency Department (ED) or who are hospitalized following a fall are at increased risk for further falls and fall-related readmissions[21–23].

Opportunistic assessment and prompt management of impaired vision in older people attending acute hospitals, following a fall, may help reduce recurrent falls risk and the associated burden on health and social care services [24]. As part of multifactorial falls risk assessment, national and international guidance recommends assessing and managing impaired vision in older adults who attend a healthcare setting following a fall [13, 25]. However, these guidelines are not being consistently implemented in practice in acute hospitals [26–28].

Survey studies and qualitative research have explored barriers and facilitators to implementing falls prevention practice guidelines and falls risk screening in acute settings, including hospital wards, outpatient clinics and ED [29–34]. Barriers reported include: limited time, resources, communication between disciplines, patient motivation and health status, staff and patient knowledge, availability of support staff and champions, lack of ownership, lack of positive reinforcement and forgetting or being complacent about guidelines [29–34]. Facilitators included: innovation appropriateness, importance and attractiveness of innovations to users, patient and staff education and training, reminders, auditing practice, feedback, champions, use of data to inform practice change, and leadership with clear goals and commitment to falls prevention.[29, 34]Barriers and facilitators to hospital vision screening for falls prevention have scarcely been studied. A survey of 49 hospital health care professionals (HCP), working across 17 Belgian geriatric medicine wards, evaluated the feasibility of implementing a multi-disciplinary practice guideline for inpatient falls prevention. This study included geriatricians, nurses, occupational therapists, and physiotherapists. Compared to other falls risk factors, including mobility impairments, medications and postural hypotension, HCPs felt vision impairment was the risk factor they felt least capable of, or responsible for, assessing and managing. [30] Due to the implementation gap in acute hospitals, the barriers and facilitators to vision screening in older people attending hospital, following a fall, need to be better understood as an initial step toward developing appropriate interventions to improve routine vision screening.

Determinant frameworks facilitate a better understanding of the factors influencing uptake of evidence-based practice in the real world [35–37]. The Consolidated Framework for Implementation Research (CFIR) is one of the most widely used frameworks for implementation research in healthcare settings [38–44], providing a structured approach to identifying barriers and facilitators to implementation. This study aimed to use CFIR to explore barriers and facilitators to vision screening in older adults attending hospital following a fall, from the perspectives of the falls Multidisciplinary Team (MDT) in an acute hospital.

Methods.

A case study approach was used to purposively recruit HCPs at a single large National Health Service (NHS) teaching hospital trust in the East Midlands of the UK. This trust includes two acute teaching hospitals. Currently vision screening is only formally mandated at either hospital for older adults admitted with a fall-related Neck of Femur fracture and this is a clinical bedside assessment conducted by orthoptists [45]. Falls risk assessments are otherwise conducted for all inpatients on healthcare of older people wards. These are performed by nursing staff on admission and 2-hourly thereafter. The assessment includes asking whether the patient has glasses and any other visual problems.

Participants were recruited via posters, staff newsletters and bulletins, and cascaded via email to relevant clinical teams. Participants were eligible if they were aged 18 years or older and involved in the care of older adults who attend hospital following a fall, in their current or previous role at the hospital.

Focus groups were organized until data saturation was reached [46]. Data saturation was determined by the identification and rich understanding of the majority of thematic issues that addressed the research aims [46–48]. Consistent with other studies with similar discussion structure and topic complexity, 4–5 focus groups with skilled facilitation were considered sufficient for this study [47, 48]. Five 60–90 min in-person focus groups were held in a hospital meeting or training room between February and June 2025. Only participants and facilitators were present at focus groups. Participants initially completed a demographic questionnaire asking about age, gender, ethnicity, highest educational qualification, current profession, years of relevant experience, last eye test, and ocular history. Vision-related data were collected to determine whether experience of vision impairments influenced HCPs perspectives on vision screening.

**Fig. 1:**
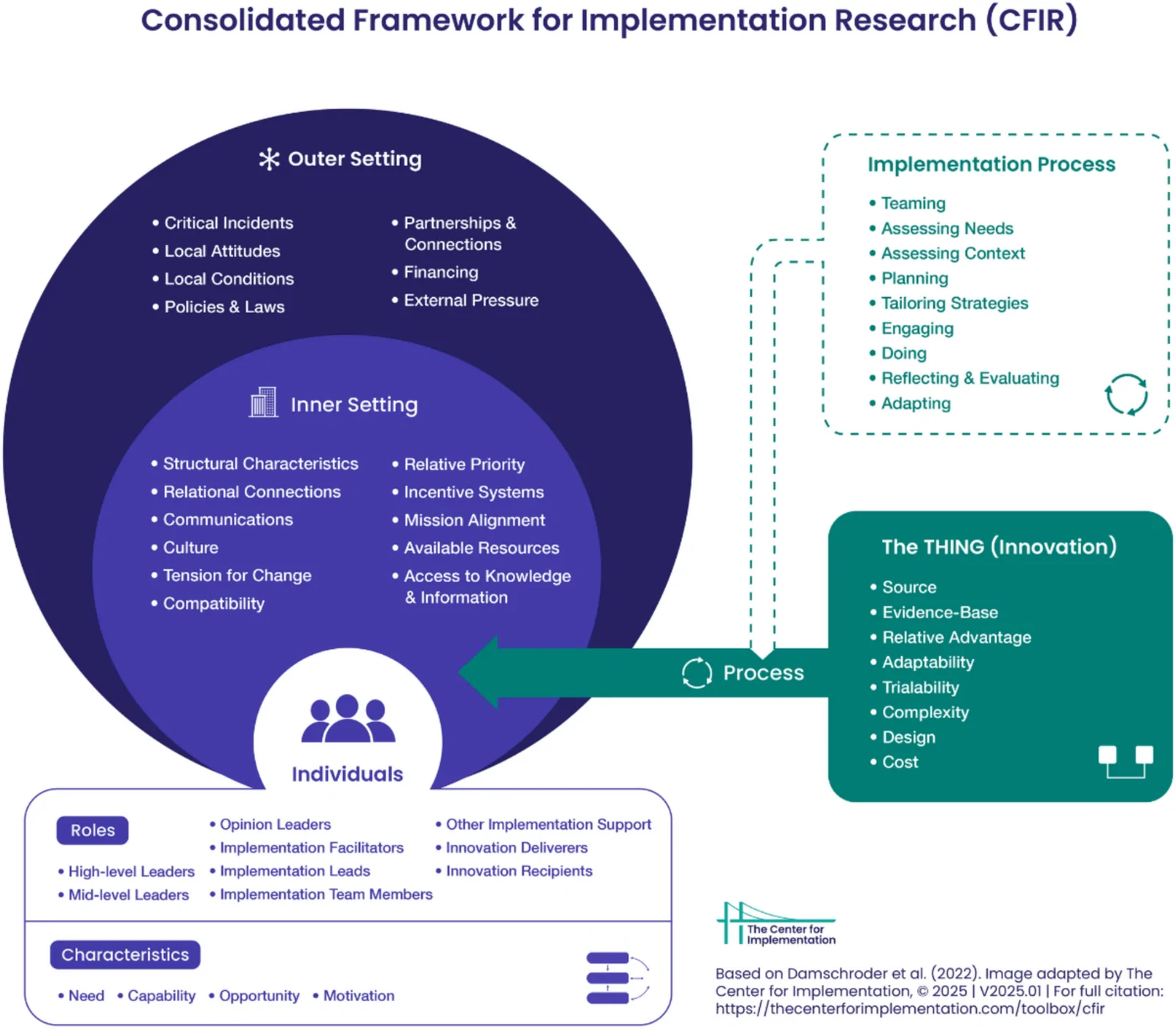
Diagram of the consolidated framework for implementation research, highlighting the 5 domains.

The CFIR is a theoretical framework to help identify barriers and facilitators to implementation. CFIR constructs are sorted into 5 domains (Individual, Inner setting, Outer setting, Innovation and Implementation process) as shown in Fig. 1.[49, 50] CFIR now incorporates the ‘Capability, Opportunity, and Motivation’ components of the COM-B Model, as constructs within the ‘Characteristics’ subdomain [49]. COM-B, which forms the basis of the ‘Behavior Change Wheel’, helps understand how human behaviors are generated through interplay between individual capability, opportunity, and motivation [51].

A semi-structured topic guide informed by CFIR supported discussions. The topic guide was reviewed by the research team, stakeholder group, patient and public involvement (PPI) advisory group and colleagues prior to use (Appendix 1). Participants discussed: the role of impaired vision in falls, the importance of assessing vision in hospital for older adults following a fall, barriers and facilitators to doing so, and the involvement of Eye Care Professionals (ECP), such as optometrists, orthoptists, ophthalmologists, and associated professionals, in falls management. Data were recorded digitally and handwritten field notes taken by facilitators.

All participants provided informed consent before participating. This study received approvals from the Health Research Authority (HRA), Health and Care Research Wales (HCRW) and the East Midlands—Nottingham 2 Research Ethics Committee and adheres to the Declaration of Helsinki. The study protocol was published on ClinicalTrials.gov, ID: NCT06645080.

### Research team and reflexivity

Author AB conducted the focus groups as part of a PhD study. AC was co-facilitator and a member of the PhD supervisory team. AC has extensive experience in focus group research, particularly in rehabilitation and dementia. Both facilitators were female, AB is an orthoptist and AC a physiotherapist. Both have shared experience working at the hospital and caring for older people following a fall. This helped create a comfortable non-judgmental environment, enabling participants to speak openly [52, 53]. KR, AG, and JM were members of the supervisory team and have expertise in mixed-methods research related to the healthcare of older people. KR is a female professor in rehabilitation research and occupational therapist by background. AG is a male professor in care of older people and a geriatrician. JM is a female lecturer in orthoptics. The team is geographically dispersed and reflect some of the professions within a falls MDT and ophthalmology.

Given that the primary facilitator and author is an orthoptist, assessing and managing impaired vision is a professional priority and she has current knowledge of how this should be done. Additionally, AB has prior experience of inpatient vision screening for older adults admitted with a fall-related Neck of Femur fracture. This experience increased the awareness of the potential effect of vision on falling and national recommendations in relation to vision and falls. This experience was also one of the motivators for this research. All team members contributed to the interpretation of data, as well as the stakeholder and PPI advisory group, to ensure data interpretation was not shaped solely by this perspective, but by a balance of perspectives.

### Data analysis

Demographic data were inputted onto Microsoft Excel and summarized using descriptive statistics. The first two focus group recordings were manually transcribed verbatim by AB. Subsequent recordings were transcribed using an automated transcription service. All transcripts were anonymized and cleaned by AB. Transcripts were not returned to participants for correcting.

Transcripts were coded inductively using thematic framework analysis, then deductively using CFIR. Inductive coding enabled data-led theme development and a deeper exploration of themes in relation to the case study. [54] Deductive coding using CFIR ensured key contextual factors influencing implementation were not missed. [49] Inductively developed themes were mapped to deductively coded CFIR constructs.

Initially, two transcripts were coded independently by AB and AC. Coding partners met to compare codes and develop a thematic framework, which was then applied to the remaining transcripts by AB [55] (Appendix 2). Themes were refined by the coding partners, research team, stakeholders, and PPI advisory group. A matrix thematically charted data, for each focus group (Appendix 3). Coding partners kept a diary of reflexive notes, which together with field notes, were integrated into the results and discussion [55]. Participants were not asked to provide feedback on findings before reporting.

This study is reported following the Consolidated criteria for Reporting Qualitative research (COREQ) [56]

## Results

### Participant characteristics

Nineteen participants were recruited, comprising two males and 17 females. Participants were aged between 18 and 54 and eight were of non-white ethnicity. They came from a range of professional backgrounds, were mostly employed in inpatient settings and had varying experience caring for older people and people who had fallen (Table 1). Eleven participants reported having an eye test in the past two years and these participants had glasses prescribed, which they wore at least sometimes. Two participants reported other ocular history, which included blepharitis and meibomian gland dysfunction.

**Table 1:** Demographics of included participants

Demographics	Participants (*n*=19)		
Gender	Female	17	(89.5%)
	Male	2	(10.5%)
Age (years)	18–24	4	(21.1%)
	25–34	4	(21.1%)
	35–44	9	(47.4%)
	45–54	2	(10.5%)
Ethnicity	White (British)	11	(57.9%)
	Asian	4	(21.1%)
	Black/African/Caribbean	3	(15.8%)
	Mixed/multiple ethnic groups	1	(0.8%)
Profession	Physiotherapist	6	(31.6%)
	Healthcare assistant	5	(26.3%)
	Nurse	3	(15.8%)
	Occupational therapist	2	(10.5%)
	Physiotherapy assistant	1	(0.8%)
	Advanced clinical nurse practitioner	1	(0.8%)
	Orthoptist	1	(0.8%)
Years of experience working with older patients	Less than a year	3	(15.8%)
	1 to 2	3	(15.8%)
	2 to 5	4	(21.1%)
	5 to 10	4	(21.1%)
	10 or more	5	(26.3%)
Years of experience working within falls MDT	Less than a year	3	(15.8%)
	1 to 2	3	(15.8%)
	2 to 5	6	(31.6%)
	5 to 10	4	(21.1%)
	10 or more	3	(15.8%)
Primary work setting	Inpatient	16	(84.2%)
	Outpatient	3	(15.8%)

*MDT* multidisciplinary team

### Thematic findings

**Fig. 2:**
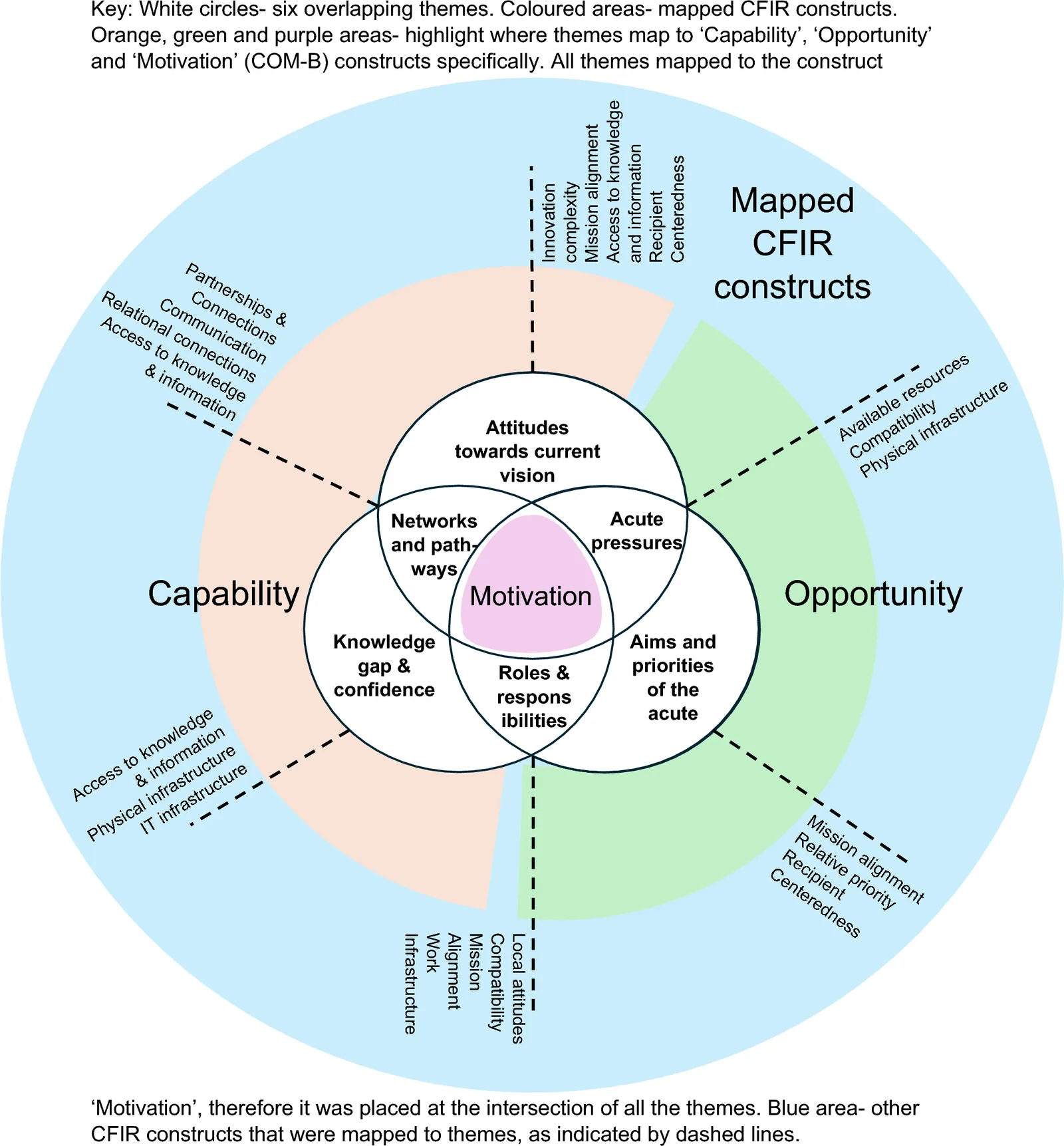
Diagram of the six inductively derived overlapping themes that were mapped to deductively coded CFIR constructs

**Fig. 3:**
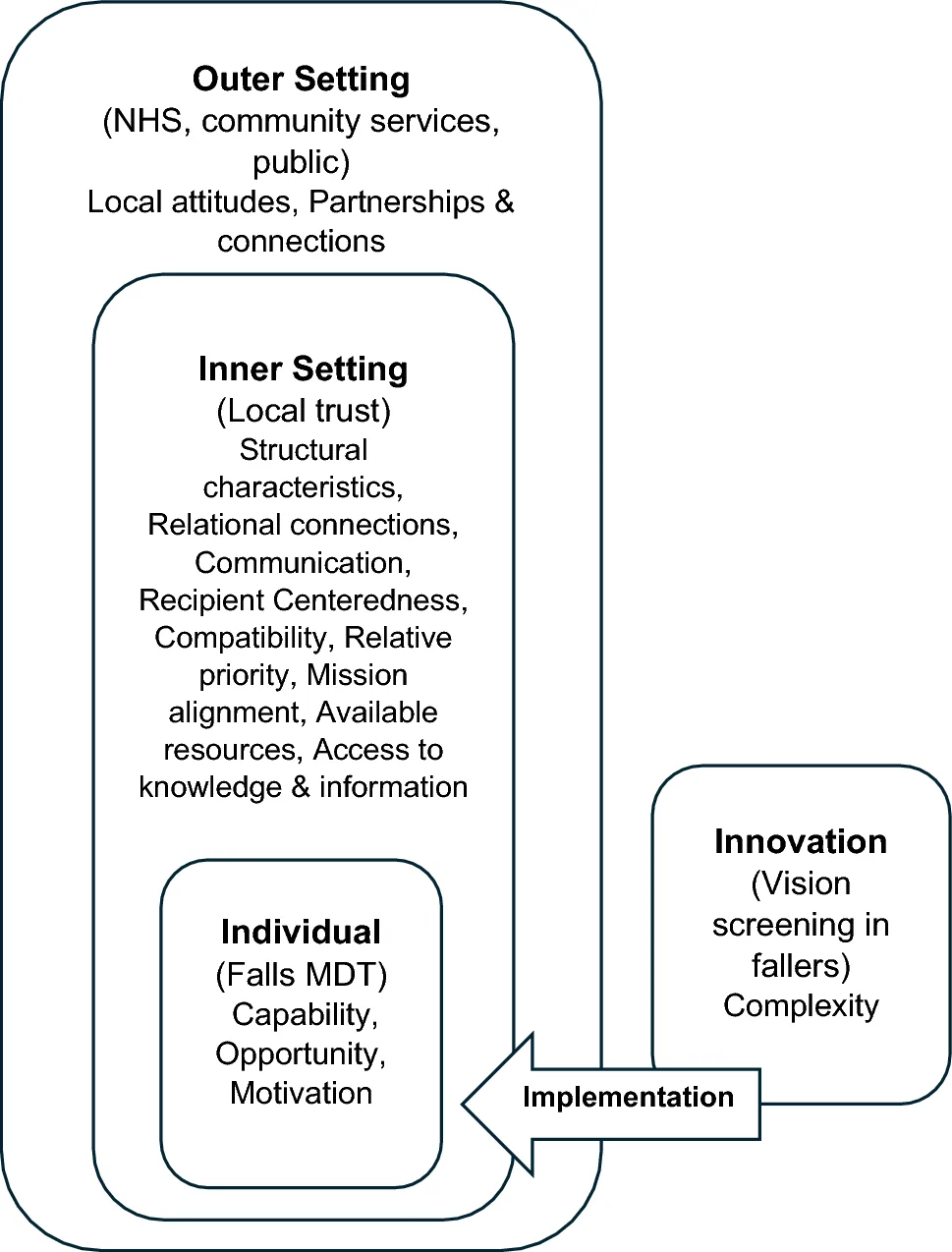
CFIR constructs coded from the data from each CFIR domain

Data saturation was reached after five focus groups. Six overlapping themes were identified, related to HCP’s views on vision screening in older adults who attend hospital following a fall. These encompassed: current practice, acute pressures, priorities in acute care, roles and responsibilities, knowledge gap and confidence, networks, and pathways (Fig. 2). Themes mapped to 14 different CFIR constructs also shown in Fig. 1, including all three COM-B components embedded as constructs within CFIR. Figure 3 shows the CFIR constructs coded from the data and the CFIR domains they belonged to. Most data were coded against constructs from the ‘Inner setting’ and ‘Individuals’ domains.

Key: White circles—six overlapping themes. Colored areas—mapped CFIR constructs. Orange, green and purple areas—highlight where themes map to ‘Capability’, ‘Opportunity’ and ‘Motivation’ (COM-B) constructs specifically. All themes mapped to the construct ‘Motivation’. Therefore, it was placed at the intersection of all the themes. Blue area—other CFIR constructs that were mapped to themes as indicated by dashed lines.

Table 2 presents the themes, sub-themes, mapped CFIR constructs, and example findings summarized from the data, highlighting the key barriers and facilitators to vision screening. Themes and mapped CFIR constructs from Fig. 2 are further described below. Additional data supporting each theme can be found in Appendix 3.

**Table 2:** Key barriers and facilitators identified from thematic findings with mapped CFIR constructs

Theme	Theme definition	Subthemes	Mapped CFIR construct	B/F	Example finding summarized from data
Networks and pathways	Networking and communication between the falls MDT and ECPs	Unclear management pathways	Partnerships and connections Communications Relational connections Opportunity Motivation	B	The falls MDT don’t know how to refer to an eye care professional if they detect a vision problem
		Unfamiliarity with eye care professionals	Access to knowledge and information Relational connections Opportunity	B	The falls MDT do not have established communication networks or points of contact with the ophthalmology department
Priorities in acute care	Individual and collective perceptions of vision screening in older adults who attend hospital following a fall and the priority of vision screening in acute care	Importance of vision in falls	Mission alignment Motivation	F	Participants agreed impaired vision was an important falls risk factor.
		Acute conditions take priority	Relative priority Opportunity Motivation	B	Impaired vision was not viewed as an acute condition. Therefore, it could be managed in the community
		Prompts to consider vision	Relative priority Opportunity	F	Incorporating a vision assessment onto existing (electronic) falls checklists would remind staff to complete it
		Importance of assessing vision in hospital	Mission alignment Motivation	F	Participants agreed that impaired vision can affect hospital rehabilitation and have knock-on effects on health outcomes
		Overprotecting inpatients	Recipient-centeredness Opportunity	B	Staff doing things for patients may make it difficult to identify those that are struggling with their vision
Knowledge gap and confidence	Limited training and confidence among HCPs in assessing vision	Training gap across staff groups	Access to knowledge and information Capability Motivation	B	Staff do not receive training on how to assess vision
		Preferred training modes		F	There are opportunities to train staff in existing departmental weekly/monthly teaching sessions
		Assessing vision in cognitively impaired patients		B	Staff felt it would be difficult to assess vision in cognitively impaired patients
		Physical resources	Physical and Information Technology Infrastructure Capability Motivation	F	A physical aid, such as an (electronic) vision screening tool would help guide staff vision assessments
Acute pressures	Working pressures in acute settings affected the opportunity of staff to assess vision in older adults who attend hospital following a fall	Equipment and space availability	Physical infrastructure Available resources Opportunity Motivation	B	Not having readily available vision assessment equipment would deter staff from assessing vision
		Time and staffing burden	Available resources Compatibility Opportunity Motivation	B	A lengthy vision assessment would be difficult to integrate into already busy job plans
Current practice	HCPs described their current practice in relation to the assessment of vision in older adults who attend hospital following a fall	Clinical impact of formal assessment	Innovation complexity Access to knowledge and information Recipient-centeredness Capability Motivation	B	Current assessments have low clinical impact as they are not thorough enough
		Informal observational assessment	Mission alignment Recipient-centeredness Capability Motivation	F	Staff detect impaired vision through observational assessments
Roles and responsibilities	Perceptions of the roles responsibilities of HCPs in the falls MDT related to vision screening in older adults who attend hospital following a fall	Roles of falls MDT	Work infrastructure Opportunity	B	No clear responsibility for assessing vision
			Work infrastructure Mission alignment Compatibility Opportunity Motivation	F	Shared responsibility would improve screening coverage
		Patient knowledge and engagement in self-care	Local attitudes Motivation	B	Patients don’t adhere to advice regarding vision

*B* barrier, *F* facilitator, *MDT* multi disciplinary team, *ECP* eye care professional, *HCP* health care professional

### Networks and pathways

This theme related to lack of networking and communication between the falls MDT and ECPs. Despite support for multi-disciplinary falls management and collaboration with ECPs, participants often lacked understanding of different ECP roles. Communication between ward-based staff and ECPs, who were predominantly outpatient-based, was limited.

*[Participant 16] “You tend to see like, the similar people, like you see people like therapies and then like SALT and dietetics. And it is. It is literally just if you see someone more often, you're gonna remember,”*

This theme crosscut the Outer setting, Inner setting, and Individual CFIR domains as participants were unsure about when and how to refer patients to hospital or community eye services. It was largely seen as the medical team’s role to liaise with ECPs if needed. Lack of clear referral criteria and management pathways for impaired vision were viewed as significant deterrents to assessing vision, rendering assessments ‘tokenistic’ without clear routes for forward referral. Clear referral criteria and a recognized point of contact were seen as facilitators to vision screening.

*[Participant 2] “…a lot of the time people aren’t comfortable with doing certain assessments because, ok if you find out there’s a deficit, now what?...when we ask questions it’s really important that that’s then relevant and followed-up and we’re not just asking questions for questions sake.”*

This theme mapped to multiple CFIR constructs, including: ‘Partnerships and Connections’, ‘Relational Connections’ and ‘Communications’. These constructs relate to internal and external networks, relationships and information sharing practices between the hospital falls MDT, and hospital and community eye services [49]. This theme also mapped to the ‘Access to Knowledge and Information’ construct, which refers to accessing guidance and training to implement practices [49], including guidance on referral criteria and management pathways for impaired vision.

### Priorities in acute care

Participants from all professional backgrounds believed vision to be an important falls risk factor, but one that did not receive enough attention in acute care. This mapped to the ‘Relative Priority’ and ‘Mission Alignment’ CFIR constructs. ‘Relative Priority’ relates to the importance given to assessing vision compared to other initiatives [49]. ’Mission Alignment’ refers to the degree to which assessing vision aligns with the perceived purpose, or goals, of the hospital falls MDT [49]. The relative priority of assessing vision in acute falls care was low compared with other assessments, such as blood pressure or medication reviews. This was said to be because impaired vision was infrequently considered to be the reason for admission or the fall, an acute condition, or one that affected hospital discharge. Managing these other conditions was considered to be more aligned with the mission of the falls MDT in an acute hospital than vision assessment.

*[Participant 19] “I'm just thinking about lots of different other aspects that might affect a patient. For example, like I said, pressure sores and things like pain or… the condition of why they're there. So, I guess vision, even though it does come in briefly. I'm thinking about lots of other things that must be done for the patient*.”

Glasses were often said to be used as a visible cue prompting staff to investigate impaired vision, or remind patients to wear their own glasses. To prompt staff to complete vision assessments, participants suggested adding vision assessments to existing mandatory falls assessment checklists.

Participants felt corrected vision could aid inpatient rehabilitation, diagnosing multifactorial causes for falls and considering unexplained deficits in other areas, such as mobility. The patient’s functional independence and quality of hospital stay could also be impacted by impaired vision. “Knock-on” effects of impaired vision included: effects on eating, drinking, taking medication, mobility and causing anxiety, which could potentially lead to malnutrition, dehydration and deconditioning.

*[Participant 4] “Cos if the vision isn’t all that great, it all has a knock-on effect, in the sense of like not being able to take the medication or malnutrition because they’re not eating or…dehydration because they’re not seeing their drink to drink it….”*

This theme also mapped to the ‘Recipient Centeredness’ construct, relating to the shared values, beliefs and norms of the falls MDT to support and care for patients [49]. Staff adapted the ward environment to protect and support patients. They ensured that objects patients needed and mobility aids were within reach and closely supervised patients when mobilizing. Staff felt more inclined to do things for patients than to give them independence, in order to keep patients safe or save time. This was also thought to be expected by the patients. Participants appreciated that this culture might make it difficult to determine which patients were struggling with impaired vision without a routine vision assessment.

*[Participant 6] “It's (vision) probably not like the first thing that comes to mind for me when I think of like… What's going to cause someone to fall? Especially like on a ward environment when people are maybe overprotected a little bit and are always walking around with someone….”*

*[Participant 8]* *“…we are making sure that all the things near their bed like the water, food, everything…”*

### Knowledge gap and confidence

Regardless of the number of years of experience or level of seniority, participants described limited training about vision. Individuals spoke of not feeling capable or confident to adequately assess vision, or manage impairments appropriately, particularly in patients with impaired cognition, who make up a significant proportion of their work. This theme mapped to CFIR’s ‘Access to ‘Knowledge and Information’ construct.

*[Participant 12] “I've been HCOP, with HCOP for 11 years now. I've never really had specific teaching on eyesight…”*

*[Participant 6] “Especially like lots of cognitive(ly impaired) patients. I don't know how I'd possibly try and do like a mini eye test.”*

Participants were keen to learn and confident that training needs could be accommodated by protected teaching time. Participants recommended resources, such as information posters for staff and patients, patient information leaflets, and an electronic vision assessment tool, to help guide assessments, incorporated into existing falls checklists. The ‘Physical and Information Technology Infrastructure’ constructs mapped here, as they capture material components or technological systems that might support vision screening in the acute hospital setting [49].

### Acute pressures

Lack of readily available vision assessment equipment, competing tasks, staff shortages, and limited time were said to affect the opportunity of staff to assess vision. This mapped to the ‘Available Resources’ and ‘Physical Infrastructure’ CFIR constructs, which together relate to the availability of physical space, materials, and equipment to support implementation of vision screening [49].

*[Participant 1] “…just getting you to do another, another thing in a list of a hundred jobs I’m tryna do”*

The ‘Compatibility’ construct, which is concerned with the fit of vision screening with current “workflows, systems and processes” [49] was also mapped to this theme. Participants reflected on the current organizational climate i.e., the limited resources of their acute setting, to suggest that a brief bedside vision assessment that does not require specialist equipment, could aid compatibility with their current ways of working.

### Current practice

Within this theme, participants described current practice in relation to the assessment of vision in older adults attending hospital following a fall. Current nursing protocols and falls checklists used by participants in this study incorporate a basic vision assessment, e.g., presence or absence of glasses. These assessments are performed 2-hourly throughout the day. Therapist assessments are not repeated but also ask broad questions about visual issues. Many participants felt assessments were unhelpful in understanding the patient’s true level of vision.

*[Participant 3] “You’re not really using that (assessment)... or actually know the truth about it (vision),”*

Some participants’ displeasure was evident in their tone, facial expressions and language, describing the assessments as *“tokenistic”,* a *“ritual”*, and *“ticking a box”*, reflecting a potential gap between formal compliance with protocols and meaningful clinical practice. The assessments were also described as “not person-centered”, which related to the ‘Mission Alignment’ and ‘Recipient-Centeredness’ constructs as person-centered care was integral to how HCPs construct their worldview [49]. The lack of depth of assessments reduced the impact on clinical practice and their person-centeredness. This mapped to the ‘Innovation Complexity’ construct, which relates to scope and degree of complexity of the vision screening assessment. Participants suggested a more in-depth vision assessment, at critical time points, such as at admission, would have more clinical impact.

Nurses, HCAs and therapists realized through discussion that they regularly performed observational assessments of functional vision and adapted the ward environment accordingly, as mentioned previously. Observations included: how steadily the patient mobilized, how accurately they reached for objects, their eye-contact and interactions with members of staff.

*[Participant 12] “But it's it's just one of those things that you don't actually think about. So, thinking about it. We actually do it on the every day, quite regularly. You don't realize it's, I don't know.”*

### Roles and responsibilities

When justifying responsibility for assessing vision in this patient group, Nurses, HCAs, and therapists in this study compared compatibility of assessing vision within their job roles and how well vision assessments aligned with their professional goals. These discussions mapped to the ‘Compatibility’ and ‘Mission Alignment’ constructs. For example, physiotherapists believed that impaired vision affected safe mobilization and was therefore their responsibility. While nurses and HCAs felt many patients may be missed due to the limited working hours of therapists and that they may have greater opportunity to assess patients. Nursing staff also felt that they see patients more regularly and build closer relationships with them, allowing them to gain a better understanding of the patient’s level of vision.

*[Participant 6]: “I think if anything it could be more, … like a therapy thing just because like in theory we're the ones saying how someone should be mobilizing. And if we think that they're not safe to mobilize a certain way because of vision, maybe that should be on us to like assess and to hand that over to like nurses and HCAs.”*

*[Participant 7]: “…as healthcare assistants you're the first ones really because you guys (therapists) might not get round to that patient…” .”*

Participants concluded that vision assessments should be a shared responsibility between staff groups, as this would improve screening coverage, reduce burden on any single staff group and be more likely to detect and manage vision problems. A simple vision assessment that could be performed by any HCP in the falls MDT was therefore suggested as a facilitator for vision screening. This theme mapped directly to CFIR’s ‘Work Infrastructure’ construct, which considers staffing levels, tasks and responsibilities of individuals and teams to support vision screening [49]. No participants mentioned the role of ECPs when discussing responsibility to vision screen.

Participants also expressed that patients should take greater responsibility for having their eyes tested in the community, reminding themselves to wear glasses and communicating their visual needs to HCPs. However, they were also aware of potential barriers to doing this.

### Mapping to COM-B constructs

Three themes (Current practice, Networks and pathways, Knowledge gap, and confidence) mapped to the ‘Capability’ component of the COM-B model. This relates to “competence, knowledge, and skills” staff need to implement vision screening [49]. The remaining three themes (Acute pressures, Priorities of acute care, Roles, and responsibilities) mapped to ‘Opportunity’, reflecting whether staff have the “availability, scope, and power” to implement vision screening [49]. The ‘Motivation’ construct refers to the drive and commitment to implement vision screening [49]. Barriers related to staff capability and opportunity, described above, affected commitment and implementation, therefore, all six themes mapped to ‘Motivation’.

## Discussion

This study aimed to use CFIR to explore barriers and facilitators to vision screening in older adults attending hospital following a fall, from the perspectives of the falls MDT in an acute hospital. The themes and CFIR constructs identified in this study highlighted three overarching discourses related to factors affecting vision screening in older adults attending hospital following a fall. First, lack of education and training on vision relevant to falls for non-ECPs. Second, the role of ECPs in the falls MDT, including lack of referral networks and integration in the team. Third, prioritization of task-focused, rather than person-centered falls care in the acute setting.

A lack of knowledge and training resulted in a lack of perceived capability and confidence in assessing and managing impaired vision among non-ECPs. This is consistent with findings of a survey of ward-based geriatricians, occupational therapists, physiotherapists and nurses who identified vision impairment as the falls risk factor they felt least capable to assess and manage [30]. Nurses, physiotherapists and occupational therapists also identified the main individual-level barrier affecting implementation of the Competence, Rehabilitation of Sight after Stroke (KROSS) visual assessment tool in Norwegian stroke rehabilitation units as perceived capability and competence [57].

Non-medical members of the falls MDT rarely communicated with the ophthalmology department and did not consider ECPs as core members of the falls MDT. This led to reluctance to assess vision as HCPs did not know how to manage and refer patients with impaired vision to ECPs appropriately. Vision assessment tools and guidelines developed by ECPs have been successful in enabling vision screening and management by non-ECPs, such as medical students, physicians, nurses, physiotherapists, occupational therapists, and assistant nurses. This includes the Visual Impairment Screening Assessment (VISA) tool for patients following stroke and brain injuries [58, 59], National Institute for Health and Care Excellence NG236 Stroke Rehabilitation in Adults guideline [60, 61], and KROSS visual assessment tool [62]. The Look out! Bedside vision check [63] was also developed collaboratively with ECPs to prevent inpatient falls, but has not yet been validated. Where capacity permits, hospital ECPs such as orthoptists have also performed vision screening in older adults who attend hospital following a fall, specifically following Neck of Femur fracture [45].

Despite not feeling capable of assessing and managing impaired vision, nursing staff and therapists in our study believed that vision screening in falls was their shared responsibility. This is in contrast to the study by Milisen et al., where participants of all professional backgrounds felt that compared to other falls risk factors, they felt least responsible for assessing and managing impaired vision [30]. This may have been related to lack of perceived capability, however reasons for feeling least responsible were not given. Participants in our study expressed how detecting and managing impaired vision in falls patients aligned with individual and organizational goals, including: ensuring patient safety, rehabilitation, discharge preparation, improving health outcomes and quality of hospital stay.

Vision screening was also considered more compatible with job plans when different staff groups worked together, as therapists may miss patients outside of their daytime working hours that nurses could capture. This echoes the findings of earlier work using the KROSS visual assessment tool in stroke units [57], where unclear responsibilities, lack of interdisciplinary collaboration to share responsibility and workload and lack of integration of ECPs in the stroke MDT, were barriers to vision screening.

HCPs were motivated to implement vision screening in falls as they felt understanding the visual status of patients aligned with their person-centered priorities of patient safety, rehabilitation, improved health outcomes and quality of hospital stay for the patient. Participants relied on observational assessments of vision and expressed dissatisfaction with formal assessments of vision that lacked depth for detecting and managing impaired vision. Participants perceived formal vision assessments as a task they had to complete in line with local protocols but that had little if any clinical impact. They described a task-focused organizational culture, due to limited resources, which prioritized the management of acute conditions, the primary reason for admission, or those affecting discharge. This presented a barrier to implementation as impaired vision did not fit these categories and created tension between commitment to person-centered care and feeling compelled to be task-focused to meet organizational demands.

Findings from the current study can inform the development of interventions and implementation strategies for improving routine vision screening in older adults attending hospital following a fall. Thus improving acute hospital management of impaired vision contributing to falls. Lack of knowledge, perceived capability and confidence was related to the ‘Access to knowledge and information’ and ‘Capability’ constructs, suggesting the need for effective training and access to relevant guidance and resources to increase staff competence, confidence and successful implementation [64–67]. Lack of referral networks, integration of ECPs into the falls MDT and undefined roles, related to multiple CFIR constructs, including ‘Mission Alignment’, ‘Compatibility’, ‘Work infrastructure’, ‘Relational connections’, and ‘Communication’. Successful implementation of innovations may be influenced by the innovation’s perceived alignment with organizational goals and a feeling of shared responsibility [68]. Implementation has also been found to be positively influenced by structural determinants, including coordination and collaboration between specialties [69], clearly defined roles [70, 71], and quality of communication within an organization [67]. These reduce confusion and empower staff with guidance and support networks. Finally, conflict between person-centered care and being task-focused due to limited resources, also related to multiple CFIR constructs, including ‘Mission Alignment’, ‘Compatibility’, ‘Recipient Centeredness’, ‘Motivation’, and ‘Available Resources’. Patient-centered care [72] has been found to be a predictor of implementation success, as has perceived mission alignment [73], as the innovation then receives buy in and commitment from staff.

In future research, our findings that mapped to COM-B components could be used with the Behavior Change Wheel to design interventions aimed at changing individual behaviors toward vision screening in older adults attending hospital following a fall [51]. Findings mapped to other CFIR constructs could be applied with compatible tools, such as the Expert Recommendations for Implementing Change for implementation strategy planning and design [40, 74]. The perspectives of the medical team and patients could also be explored in further qualitative research, to ensure all relevant stakeholders are involved for successful implementation planning [75].

The key strengths of this study related to the combined inductive and deductive coding using CFIR, which enabled a comprehensive approach to data analysis [54]. A case study approach, meanwhile, supported in-depth understanding of barriers in the acute hospital context [76, 77], making findings transferable to similar settings. Group heterogeneity and small group sizes were also a strength of this study. Our participants were heterogenous in professional background, years of experience, age and ethnicity, reflecting the mix of HCPs involved in the care of older adults. This added richness to the data and allowed a more holistic approach to the subject. As with Dahlin-Ivanoff, we found that small heterogenous groups were dynamic and easier to manage contributions of participants [78, 79].

Key limitations of the study relate to the findings coming from a single hospital, which is likely to limit generalizability and the fact that we weren’t able to recruit from the full multi-disciplinary team, particularly our inability to recruit any doctors to the study. Given their role in referral authority, priority setting, clinical leadership and overall responsibility for patient care, the absence of doctor participation could have influenced the findings in relation to acute priorities, roles and responsibilities, networks and pathways. While their perspectives would have been useful, medical staff may have introduced a power imbalance and influenced freedom of expression. Although nurses and allied health professionals may be more likely to perform vision screening, medical leadership, support and involvement in management pathways, or lack thereof could influence implementation feasibility and uptake of this in practice.

## Conclusion

Focus groups with HCPs identified barriers and facilitators to vision screening in older adults attending acute hospitals following a fall. HCPs were motivated to vision screen and felt it aligned with person-centered falls care. However, individual and contextual barriers related to staff capability and opportunity in the acute setting affected implementation. There is a need for multi-component and multi-level interventions and implementation strategies to target individual-, structural-, and organization-level barriers, including integrating ECPs into the falls MDT, engaging supportive leaders, developing an effective vision screening assessment with accompanying guidance, defined roles, responsibilities and management pathways, individual training and time allocation for staff to perform screening.

## Supplementary Information

Below is the link to the electronic supplementary material.

Supplementary file1 (DOCX 23 KB)

Supplementary file2 (DOCX 31KB)

Supplementary file3 (DOCX 60KB)

## Data Availability

The authors confirm that the data supporting the findings of this study are available within the article and/or its appendices.
