# Supplementary material for: Health care professional perspectives on vision screening in older adults who attend hospital following a fall: a focus group study
Source: Eur Geriatr Med. 2026 May 14;17(4):2247–61. doi: 10.1007/s41999-026-01486-y (PMC13562136; doi:10.1007/s41999-026-01486-y)
Supplement: Supplementary file 1 [file 41999_2026_1486_MOESM1_ESM.docx]

**Topic guide questions**

Introductory questions:

1. Is everyone happy to introduce themselves and tell us a bit about your role in caring for patients following a fall?

Opening discussion questions:

2. What do you **understand about the role** of vision in falls?
3. How **important do you feel the role** of vision is in falls, (**compared to** other factors/ health problems)?

Core questions

4. Do you **think it is important** for this group of patients to have their vision assessed as part of their care ***whilst* *in hospital***?
5. The NICE guidance recommends the assessment and management of impaired vision in older adults who attend hospital following a fall (including those hospitalised and those discharged from ED). However, these recommendations are not being implemented consistently, or in a standardised way. From your experience, why do you think that might be **(barriers)**?
   - Prompts: Taken relevant constructs from CFIR
   - Innovation domain (the national recommendations)
     - Complexity- clarity
     - Design- usability
   - Outer setting domain (hospital system)
     - Large-scale/ unanticipated events disrupt delivery?
     - Local conditions (e.g. economic, environmental, political, technological) enable outer setting to support delivery
     - Inner setting has correct external networks to deliver (e.g. referrals falls clinic > ophthalmology)
     - Sufficient legislation, professional group guidelines and recommendations, standards to support delivery
     - Sufficient external pressure driving delivery (e.g. societal)
   - Inner setting domain (ward/ clinic)
     - Infrastructure, including work infrastructure- Organization of tasks and responsibilities/ staffing
     - Networks and communication with correct teams (i.e. ophthalmology)
     - Culture- shared values, beliefs and norms regarding the delivery of the vision assessment
     - Compatibility with workflow, systems, processes, time
     - Relative priority- (perceived) importance of vision assessment
     - Mission alignment- (perceived) alignment of delivering vision assessment with setting goals
     - Available resources- funding, space, materials, staff
     - Access to knowledge/ information- guidance/ training to deliver vision assessment
   - Individuals domain
     - Leaders involved- (authority, influence, implementation facilitators aware and involved sufficiently)
     - Innovation deliverers- people who can test vision
     - Capability of deliverers- knowledge and skills to deliver
     - Opportunity of deliverers- availability, scope and power to deliver
     - Motivation of deliverers- committed to deliver
   - Implementation process domain
     - Teaming- coordination and collaboration
     - Planning- identifying roles and responsibilities, steps and milestones, goals, measures
6. What form of **intervention** do you think would help facilitate implementation of the guidance? /If you were to develop an intervention to help facilitate vision screening in this population, what would it be (considering those hospitalised and those discharged from ED)?
   - Prompts:

See above

Closing questions:

7. What do you think about eye health professionals being more involved in the falls MDT?
   - In what way do you think they could best support the falls team?
8. That’s all the questions I would like to ask, is there anything else you would like to add/ think I could have asked?
