# Supplementary material for: Health care professional perspectives on vision screening in older adults who attend hospital following a fall: a focus group study
Source: Eur Geriatr Med. 2026 May 14;17(4):2247–61. doi: 10.1007/s41999-026-01486-y (PMC13562136; doi:10.1007/s41999-026-01486-y)
Supplement: Supplementary file 2 [file 41999_2026_1486_MOESM2_ESM.docx]

| **Thematic framework** | | |
| --- | --- | --- |
| **Codes** | **Subthemes** | **Themes** |
| - Being unsure how to refer to/ contact eye specialists (in hospital or community) - Only the medical team liaise with/ refer to ophthalmology - Uncertainty of outcome of community vision assessments - Eye specialists refusing to assess - Need for clear referral criteria - Make red flags very clear - Need for clear referral pathways - Don’t want to inundate or waste eye specialists time with inappropriate referrals - Importance of knowing how to manage and not just assess - Lack of confidence to manage - Reluctant to screen due to inability to manage | Unclear management pathways | Networks and pathways |
| - Don’t come into contact with eye specialists on the ward - Lack of involvement with the eye department - Eye specialists feel unrecognised - Being unsure about the roles of different eye specialists | Unfamiliarity with eye care professionals |  |
| - Impaired vision is prevalent amongst older people in hospital - Impaired vision is a normal part of aging - Falling in low light conditions. - Falling at night (when going to the toilet). - Reaching for mistaken objects/ lack of depth perception leading to imbalance. - Bumping into things they can’t see - Incorrect use of glasses leading to imbalance and falls. - Unexpected situations due to impaired vision - Vision being the most important of the senses in balance and falls. - Falls are mainly physical/ mechanical in nature, not sensorial. - Multifactorial nature of falls. - Vision not often seen as causal falls risk factor - Vision thought to play a significant role in falls - Vision linked to falls via mobility - Vision thought to play a big role in mobility - Impaired vision is not often thought about as a falls risk factor. - Impaired vision is not often talked about in practice. - Increased awareness and understanding can make it higher priority. - Lack of attention to vision is a barrier to vision assessment | Importance of vision in falls | Priorities in acute care |
| - Vision overlooked in the presence of other life-threatening conditions - May not take notice of vision on admission, but may be considered later - Focussed on what keeps patient in hospital and what aids discharge. - Task-oriented rather than patient-oriented. - Impaired vision not considered an acute condition. - Medical emergencies or observable harm take precedence - Vision not usually reason for admission so not priority - Conditions that give the patient pain take precedence - Unclear how much impact glasses have with other compounding factors e.g. low blood pressure - Need to detect visual changes during hospital stay - New visual issues should be assessed in hospital vs longstanding - Really impactful visual issues should be assessed in hospital | Acute conditions take priority |  |
| - Signs above bed indicating vision impairment prompt - Prompted to consider vision only if known severe condition - Observable harm prompts investigations - Glasses can prompt staff to ask about/consider vision - Staff prompt patients to wear glasses only if visible - Vision assessments should be incorporated into existing falls assessments - Making it part of mandatory assessments may help - Add vision assessment to existing electronic assessments - To have vision screening tool on nerve centre would be beneficial | Prompts to consider vision |  |
| - Can tailor rehabilitation activities - Need to know for safe rehabilitation activities - Need to know if need glasses for safe mobility - Affects engagement in independent tasks - Help to determine cause of fall - Help reduce hospital falls risk - Considering what the patient would want assessed and managed - More likely to keep patient at home if poor vision as familiar environment - Need to know appropriate aids for discharge - Need to know what to advise patient regarding vision on discharge - Affects quality of hospital stay - Anxiety in hospital environment - Feeling lonely - Crying and yelling - Affects confidence to mobilise - Will struggle to function as hospital environment is unfamiliar compared to home environment - Impaired vision has knock-on effects - Affects eating - Affects drinking - Affects mobilising - Affects ability to see and take medications - May lead to deconditioning - Staff felt patients need to be wearing correct glasses for correct task | Importance of assessing vision in hospital |  |
| - Hypervigilance on wards due to falls risk - Proud of proactive care and close supervision of patients - Overprotectiveness may not be good falls prevention strategy - Toilet supervision - Walking with patients - Ensuring they have the right mobility aids - Rearranging bed space from what patient might want - Bringing things closer to patients and their bed - Removing clutter - Think we are being helpful by moving things but may not be - Doing things for patients - Overprotection may not be beneficial for patient independence - Unable to assess level of vision if doing things for patients | Overprotecting inpatients |  |
| - Lack of current training provision for nursing or therapies on vision - Eye specialists don’t get training on falls either - Some aware other visual functions than visual acuity to assess, but don’t know how - Don’t feel capable to do more than tick-box assessment - Lack of MDT knowledge on vision in general - Don’t know how to tell if patients are wearing the correct or most up-to-date glasses - Lack of education on glasses types and usage for staff and patients - Confusion more likely to be considered than vision as better understood - Keen for training. - Lack of knowledge to manage - Want to know what they can offer patients without referral, if referral post discharge - Education can raise awareness of different eye specialists - Understanding need and benefit could outweigh time burden - There should be local guidelines and protocols - Some unsure if there were existing guidelines and protocols - Forgetting guideline content - Lack of confidence in findings | Training gap across staff groups | Knowledge gap and confidence |
| - Understanding lived experience useful in training, by simulation - Could be part of mandatory, rotational training, or self-directed. - Regular departmental teaching sessions available - Teaching part of job plan - Incorporate in training of other conditions that affect vision, such as diabetes and falls. - Should teaching be provided by specialist or non-specialist. | Preferred training modes |  |
| - Dementia - Confusion - Acute illness - Delirium - Don’t know how to approach vision assessment with impaired cognition - Different type of assessment equipment needed (impractical) - Not right environment for testing these patients - Can’t rely on accurate history - May not be aware of vision problems - Relatives can give information - Patients may not have the patience for assessment - Patients may get agitated for assessment | Assessing vision in cognitively impaired patients |  |
| - Screening assessment tool would be useful - Prefer to have a physical resource to guide - Need for signposting resources for staff and patients (posters, numbers) - Awareness of bedside vision tool, but not used | Physical resources |  |
| - Difficulty replacing or finding glasses - Glasses not being available in emergency admissions - Relatives may take glasses home in case they get lost or damaged in hospital - Lack of availability of vision assessment equipment on ward - Limited time to find or print resources - Accessibility of relevant forms to fill out - Ideally no specialist equipment should be required for screening (availability) - Need bedside assessment - Assessment shouldn’t require special space if to be done on ward | Equipment and space availability | Acute pressures |
| - Multiple competing tasks - Competing with other staff and their tasks to assess vision - Quicker to do things for patients than consider vision - Time feasibility of assessment depends on content of assessment - Education may not be enough to promote screening due to time burden - Too many patients to screen is a barrier - Not feasible to assess all older patients - Target specific population to vision screen otherwise too many - Brief vision assessment favourable - RCP guidance currently too long - May be better in admitted patients rather than discharged due to time - May be better done in outpatient setting (more time) | Time and staffing burden |  |
| - Two-hourly nursing assessment of glasses - Asks broadly about visual history on proforma (therapists) - Sometimes asking patients about visual symptoms beyond protocols - Sometimes asking patients about their glasses and use of glasses beyond protocols - May require probing, but relies on initiative of staff - Formal assessment useful as a reference when seeing many patients - Asking relatives - Looking at medical notes - Nursing handover - Becomes ritualistic - Feeling of ‘ticking a box’ - Tokenism in current assessments - Doesn’t feel patient-centred - Assessment should not be too frequent - Assessment should be done at strategic time points during hospital stay - Assessments seem to be done for defensive practice and litigation - Assessment on admission would be most helpful - Assessment before patient discharged may also be helpful - Reminders and prompts are useful - Very frequent assessments lose clinical power and don’t affect clinical practice - Questioning accuracy of frequent checklist assessments   - Lack of clinical reasoning involved - Automatic responses that are not actually assessed - Depth of assessment needs to be appropriate for acute setting - Absence/ presence of glasses too simplistic for assessment of vision - Current assessment protocols are too basic - Visual function in hospital may not reflect function at home - More in-depth assessment may be useful for giving Drs evidence of impairment - If just relying on patient history, may not always be reliable | Clinical impact of formal assessment | Current practice |
| - Don’t realise they are assessing vision this way (on a daily basis) - Mistaking objects - Being unable to see a distance object pointed to - Misjudging depth of objects - Lack of spatial awareness - Reaching inaccurately - Missing things - Bumping into things - Assess stability of mobility - Over-shooting with walking aids - Trouble with eating and drinking - Reading newspaper - Using their phones - Struggling to read the clock - Poor eye contact - Being startled by staff - Not following with eyes - Closing the eyes due to uncomfortable vision | Informal observational assessment |  |
| - Eye specialists could be within a falls MDT - Multidisciplinary working enables better picture of patient condition - Therapist have skills in assessing visual field in stroke patients - Therapists role as determining who mobilises - Therapists limited hours so may miss patients - Nursing/ HCA provide 24hour care so can capture more patients - HCAs see more of what patient can and can’t do - All staff need to be more inquisitive - Some staff don’t remember to complete assessments as per protocols - Everyone’s responsibility - Any staff member who sees the patient first - Shared workload as busy jobs - If all staff involved can get holistic picture of visual ability - Nursing and therapies responsibility to assess, then refer to Dr - Depends on skills required to assess - Lack of clarity on whose responsibility it is - Difficulty recruiting rotational staff to screen | Roles of falls MDT | Roles and responsibilities |
| - Patients should have regular eye tests in the community - Patients wearing old glasses - Patients having eye tests many years ago - Glasses often dirty - Cleaning patients glasses - Staff/ experts don’t have regular eye tests themselves - Barriers to involvement: - Cognition - Mobility - Transport - Being housebound - Relying on relatives - Cognition - Costs - Confidence - More urgent health problems - Too many appointments to attend - Accepting nothing to be done about poor vision with age - Managing fine at home in familiar environment - Not prioritised by patient - Adapt to impaired vision and may not realise how bad it is - Patients don’t complain about impaired vision in hospital unless changing - Patients don’t want to disturb staff - Patients not communicating visual issues or needs to staff - Patient may tell you when you ask them to do something they can’t do - Patients need reminding to wear their glasses - Patients don’t think of wearing their glasses or turning the light on - Patients relying on staff when in hospital - Due to health, may not be able to go to optician on discharge - Can’t rely on patients to see optician on discharge - Staff don’t take any responsibility if asking patient to see optician on discharge | Patient knowledge and engagement in self-care |  |
