# Supplementary material for: Health care professional perspectives on vision screening in older adults who attend hospital following a fall: a focus group study
Source: Eur Geriatr Med. 2026 May 14;17(4):2247–61. doi: 10.1007/s41999-026-01486-y (PMC13562136; doi:10.1007/s41999-026-01486-y)
Supplement: Supplementary file 3 [file 41999_2026_1486_MOESM3_ESM.docx]

| **Thematically charted example data for each focus group** | | | | | |
| --- | --- | --- | --- | --- | --- |
| **Focus Group number** | **FG1** | **FG2** | **FG3** | **FG4** | **FG5** |
| **Group composition** | **P,HCA,OT,HCA,N** | **P,HCA,N,HCA** | **P,PA,ACP** | **OR,P** | **P,OT,HCA,SN,P** |
| Themes | Subthemes with example data | | | | |
| Networks and pathways | Unclear management pathways  PARTICIPANT 2: “…its really important that that’s then relevant and followed-up and we’re not just asking questions for questions sake,”  Unfamiliarity with eye specialists  PARTICIPANT 2: “I was trying to get a referral to orthoptics, no one on the ward knew who they were as a profession.” | Unclear management pathways  PARTICIPANT 9: “And then I'll mention with the doctors as well to see if this patient could be referred to the ophthalmologist… But I don't think I've come across such.”  PARTICIPANT 6: “I wouldn't know what to try and do for like community follow up,”  PARTICIPANT 6: “I'm guessing that the way to reach the like the eye department wouldn't be too tricky now, I think it's just that no one's ever like, explained how to do it.”  PARTICIPANT 6: “Yeah. Well, it's just like if I understood the services that we could get involved better, I'd be more inclined to to try and like work it out.”  PARTICIPANT 6: “…to work out like at what point do they meet the criteria or actually like they need more care than just this can be managed and like they're going to be changes as you get older?”  PARTICIPANT 6: “…if you're going to tick one of those and you know like this is an appropriate referral. If you don't have something like that, then you're kind of just guessing, like if whether or not they actually need it.”  PARTICIPANT 6:“…you'd end up having loads and loads of referrals because it's quite tricky to like if it's yeah, I don't know how I could like distinguish within my skill set of if it's like a something they can manage or not.”  Unfamiliarity with eye specialists  PARTICIPANT 7: “We don’t really have any involvement in the eye department.” | Unclear management pathways  PARTICIPANT 10: “But I I don't even know if I've got anyone for vision.”  PARTICIPANT 10: “I have seen a couple of patients having their eyes tested on the ward before. But that was a very long time. I don't know what the referral process is now for it, but I'm assuming that's nerve centre.”  PARTICIPANT 11: “So we regularly send our patients around to eye casualty, but that's for like acute eyesight or issues like that you think there's a problem like looking at the back of the eye…”  PARTICIPANT 11: “How do I say this PC, we sometimes refer patients to eye casualty and then sometimes we’re told no don't send that patient to eye casualty… Who exactly is it that they're going to want to see?”  PARTICIPANT 12: “Is there a way of us doing a referral to their opticians or?”  Unfamiliarity with eye specialists  PARTICIPANT 11: “My daughter had squint surgery in the summer. And I still have no idea who's who. I don't. I don’t know, I don’t. Orthoptists. No offence, I’ve got no idea.” | Unclear management pathways  PARTICIPANT 13: “If their contact’s on there (nerve centre). Then I feel like it would be pretty easy from there. But if not. Then probably wouldn't be very easy no I don't think so. Unless I knew someone who knew their contact. Like speaking through people.”  PARTICIPANT 13: “I'll always speak to the doctors about it or say. Do you guys know who can refer on and then it’s kind of left with the doctors slash if there's anyone on nerve centre who I can contact?”  PARTICIPANT 14: “It (vision) can't be managed by the ward staff, ward staff, the doctors can't fix it, can they?”  PARTICIPANT 13: “I feel a bit out of my depth with like being able to do anything about it?”  PARTICIPANT 14: “…there be no good testing them without them being able to act on it”  Unfamiliarity with eye specialists  PARTICIPANT 14: “I chitchat to people I see a lot on the ward. And they know what I'm there to do. But that's really the people that I see in passing and generally when the doctors change over, people are unaware of what I'm doing and I have to tell them.”  PARTICIPANT 13: “…there's so many people on my ward who have, like, vision problems who I think would benefit” (from input from eye specialists)  PARTICIPANT 14: “…no one knows who you are necessarily. You kind of feel that what everyone else is doing (on the ward) matters that little bit more and it doesn't, but I don't know it can do can't it,”  PARTICIPANT 14: “I think it's important (assessing vision). I think it matters and I think it's just a shame that nobody else seems to appreciate that and that they don't. Yeah, I just get on with it. I think it's frustrating to have to explain to the new doctors each time what we're doing, and they all seem quite delighted that it's happening. But it's a shame that nobody else tells them that it's happening, because then we could be a resource for them as well.” | Unclear management pathways  PARTICIPANT 17: “So we'd mention it to our medical team and in our ward rounds…”  PARTICIPANT 17: “And don't know if it would be my role to liaise directly with another specialty like the eye, eye ophthalmology for a new new identified problem. I'd expect the doctors to go in and do that more in depth assessment…”  PARTICIPANT 17: “I think the nurse she or he would probably refer to Doctor who might call the specialist or might refer themselves.”  Unfamiliarity with eye specialists  PARTICIPANT 19: “I don't think I do personally. (know the difference between eye specialists). Obviously I’m now gonna go back and find out.  Moderator 1:  Ok yeah. Anybody else? All right. (heads shake) Don’t worry, this is not uncommon.”  PARTICIPANT 16: “I guess because we're all ward based. You tend to see like, the similar people like you see people like therapies and then like SALT and dietetics. And it is. It is literally just if you see someone more often, you're gonna remember, you’re gonna maybe chat to them more.“ |
| Priorities in acute care | Importance of vision in falls  PARTICIPANT 5: “…they don’t think of wearing glasses or turning on the light, so, I think the falls are very common during nights.”  Acute conditions take priority  Vision was considered one of the main senses associated with falls:  PARTICIPANT 2: “…main ones when you think of falls risk”.  “(vision) falls by the wayside”.  PARTICIPANT 1: “…people just don’t know because we don’t talk about IT,”  PARTICIPANT 1: “…those things that seem like an actual could be a medical emergency” take precedence.  PARTICIPANT 1: “Yh because if you can see someone bleeding that’s a problem, if you can see their blood pressure and they’ve gone all dizzy that’s a problem, whereas like vision, its harder isn’t it, you cant quite think of it as such, connect with it,”  Prompts to consider vision  PARTICIPANT 1: “…ideally what you’d want is that almost like, so on nervecentre when you do all your different things like your must score and all of those you’d kind of want something on there,”  Importance of assessing vision in hospital  PARTICIPANT 1: “…get a bit of a better understanding of anything that we need to put in place,”  PARTICIPANT 1: “…engage in independent tasks as much as possible and their mobility”.  PARTICIPANT 1: (Managing vision helps) “…to actually make it a good hospital stay as well.”  PARTICIPANT 3: “Cos if the vision isn’t all that great, it all has a knock on effect, in the sense of like not being able to take the medication or malnutrition because they’re not eating or saying all dehydration because they’re not seeing their drink to drink it or, so yh, so poor vision I do believe has a knock on effect on other aspects as well  PARTICIPANT 2: What about continence?  Moderator 1: Yh  PARTICIPANT 2: If they cant see where the toilet is  PARTICIPANT 4: I think it makes them decondition because we’re not gunna walk them to the toilet.  PARTICIPANT 1: …at home, they’re probably able to have a bit more independence cos they know their own environment.”  PARTICIPANT 5: “Theres a patient in a side room and keeps shouting for help, oh am I left alone in the room, every few, like every few mins they keep calling  PARTICIPANT 3: Yh cos he is hard of, his vision’s poor. Yh think he’s just  PARTICIPANT 5: Am I left alone? He’s, he’s very anxious” | Importance of vision in falls  PARTICIPANT 8: “I feel like that's not because of the vision or sight or anything. It's sometime it's slipping from the bed or slipping from the chair or in the just missing in the toilets and all things”.  PARTICIPANT 7: “…they need to see where they're going. So if, if they've got blurred vision and they, they’re walking and they're bumping into things, they've got risk of falling over things.”  PARTICIPANT 6: “…patients won't really notice their obstacles…”  Acute conditions take priority  PARTICIPANT 9: “And we think about every other thing, but not really much about vision.”  Prompts to consider vision  PARTICIPANT 9: “So you may be kind of reminding healthcare professionals or prompting them to always check…”  PARTICIPANT 6: “No, I'd say unless if I like see them (glasses) on their table in front of them that prompts me.”  PARTICIPANT 7: “But there's the odd chance that people just put them in the drawer or leave them in the bag. So then you don't know the glasses are there.”  PARTICIPANT 9: “…it can be added to the checklist it can remind healthcare professionals too”  Overprotective of inpatients  PARTICIPANT 6: “It's probably not like the first thing that comes to mind for me when I think of like, what are the high? What's going to cause someone to fall? Especially like on a ward environment when people are maybe overprotected a little bit and are always walking around with someone that they need to, and it's like kind of helping them to to avoid that, at least in an inpatient setting.”  PARTICIPANT 9: “Make sure that they have their mobility, the right mobility aid to walk with. We make sure that they're always with someone, Someone is always with them, especially when they're at risk of falling in the toilet or something.”  PARTICIPANT 8: “And also we are making sure that all the things near their bed like the water, food, everything near their bed in the on the table and the buzzer always on the bed and the chair always near the bed.”  PARTICIPANT 8: “…always the staff is there around, so they (patients) will not complain about their eyes.” | Importance of vision in falls  PARTICIPANT 11: “If they normally wear glasses, if they've got them with them or or haven't got them with them, if they're registered blind or not like so, what is the normal level of vision and the lighting that could affect vision.”  PARTICIPANT 10: “They actually can't see properly and that may could have been a factor of falling.”  Acute conditions take priority  PARTICIPANT 11: “It wouldn't be something that I would typically ask unless it is something relevant to what a patient come in with like, if they've come in with a headache or say you know any vision changes,”  PARTICIPANT 11: “So when we've got somebody that's come in with like giant cell arteritis and they say to me I can't see out my left eye anymore then it's priority, but from me. Basic day-to-day. Less so, for us it wouldn't be something that we take huge amounts of notice of. Unless somebody said Ohh. I've come in with a headache. My vision is really blurry. Or like from an acute perspective. But if it's something long standing. We would ignore it and be it and just advise them oh you need to go get your eyes tested.”  PARTICIPANT 11: “Probably acknowledging an awareness that it needs doing.“ (is a barrier to assessing vision)  PA: “It's more confusion that registers to us it's not as much is it their eyesight. So yeah, say eyesight is not an immediate.”  PARTICIPANT 11: “Can't really say whether it helped or not, because he was still his blood pressure still dropped. So yeah, but obviously it's not gonna help if he hasn't got his glasses on.”  Prompts to consider vision  PARTICIPANT 10: “And then I'll see them (a patient) like a few days down the line. To then remember whether they're wearing glasses or not after I've seen a hundred other patients, usually what triggers me to think ohh they need to wear the glasses is when they're on the table so.”  PARTICIPANT 11: “I wouldn't think to have thought about their vision particularly unless it was something obvious like they are, you know, that we know that their vision is really quite poor or their glasses are on the table and the, you know. That would be like my prompt to think about it, otherwise it wouldn't be something that I would think they fell because of their vision.”  Importance of assessing vision in hospital  PARTICIPANT 11: “But having three catastrophic Falls, 2 resulting directly in death. Yeah, like at the time, if we can save one life it's important.”  PA: “…it's quite helpful and I've started putting which one (glasses type the patient wears) because it is quite helpful if they’re short sighted and it explains a lot as well, if they're not wearing the glasses.”  Overprotective of inpatients  PARTICIPANT 11: “If somebody is visually impaired, within their home environment, they know exactly where things are at the other place where they're expected to be placed and and you know, they may have arranged their bed space as they wish to be. But you know, us as staff might have gone in and moved things, thinking that we're perhaps being helpful, but maybe we're we're not.”  PARTICIPANT 10: “I think a lot of the time when patients come into hospital and they lose their independence, they rely on us to tell them or do things for them. So then you're then not able to assess it (vision impairment).” | Importance of vision in falls  PARTICIPANT 13: “It has a big part to play. Yeah, I often find that with my patients, if they've not got, I guess, perfect vision or they've had a change in their vision, it increases their risk of falls.”  Acute conditions take priority  PARTICIPANT 14: “It's easy to overlook it (vision) as well I think when they're coming in and they're having major surgery and this that and the other, there are times when I'm seeing a patient who's tells me that they're sight impaired they've, I've gone for a chat and they tell me. It's not written on their board.”  PARTICIPANT 13: “I definitely don’t think it’s taken seriously. Well, as seriously as like it probably should…That wouldn't stop someone from being discharged, whereas like blood pressure would, like it kind of like things like that seem a bit higher than vision but they both cause the same thing (falls).”  Importance of assessing vision in hospital  PARTICIPANT 13: “I can't get them to their functional baseline because they can’t see like they used to before. So then they can't be sent home, but not from the point of like their vision, it would be because oh they’re not therapy fit.” | Importance of vision in falls  PARTICIPANT 19: “…if you can’t see obviously you can run into obstacles… They don’t know where people’s hands are and they don’t know how to use the equipment they’ve got. To summarise, it’s really important.”  Vision was described as the:  PARTICIPANT 18: “starting point of our action.”  Compared to longstanding vision problems, it was felt that:  PARTICIPANT 19: “…the risk (of falls) will be higher”  Unsure why vision isn’t assessed but:  PARTICIPANT 16: “…if someone comes in and has a fall, they often do like lying, standing blood pressure, even if they like tripped over something,”  Acute conditions take priority  PARTICIPANT 19: “I'm just thinking about lots of different other aspects that might affect a patient. For example, like I said pressure sores and things like pain or what, what, what why they’re there, the condition of why they're there. So I guess vision, even though it does come in. Briefly. I'm thinking about lots of other things that must be done for the patient. Yeah, that's not a good thing…”  PARTICIPANT 17: “Maybe because it's not thought to be the main reason or cause of the fall…”  PARTICIPANT 19: “So if it’s fall, they fall and have a fracture. That's a bit different. But if it’s something like they have that have hypertension or heart attack or something like that. Kind of more focused on that I suppose.”  PARTICIPANT 15: “…the physio comes say oh did you realise she had poor vision? But for your first admission, you don't check that.”  Prompts to consider vision  PARTICIPANT 15: “But if you don't wear the glasses, I'm not asking you why you're not wearing the glasses.”  PARTICIPANT 15: “…when you find the glass on the table, you checked that, but when the patient is in and you don't see the glasses, you're not going to ask them.”  PARTICIPANT 19: “And then if it’s (vision assessment tool) on nerve centre. That’s even better.”  PARTICIPANT 19: “So maybe on admission could be, on the checklist could be some sort of vision assessment.”  Importance of assessing vision in hospital  PARTICIPANT 19: “Also, if you’re vision is impaired, your confidence isn’t as high, I've seen many patients who are vision impaired and so scared to actually stand up.”  PARTICIPANT 17: “So we'd refer to it (vision information) and then we'd work that into our assessment and take note of if it has an impact or or not… and maybe we'll do some specific assessments around mobilising and and kind of undertaking activities they need to, bearing in mind specifically what they can see and can't see.”  PARTICIPANT 16: “We look at discharge quite a lot and if someone's not managing because of like a new visual issue, then the kind of if that's the issue, then it kind of needs to be reviewed before we can make a plan of where they're going to go or like what they're going to do, how much help they're going to need.”  PARTICIPANT 17: “If it's something really impactful. For one another reason then? Yes, we should be doing an inpatient (vision) assessment.”  PARTICIPANT 17: “…if it's a more serious thing, then yes, it needs addressing for that patient at that point in time.”  PARTICIPANT 17: “If I was a patient, then I came into hospital and my vision was now having an impact on me being able to do things and I was, and this was a new problem or a worsening problem. I would want it looked up there and then, yes.”  PARTICIPANT 18: “Making sure they have all the necessary device, just like the eyeglasses, because we are mobilising.” |
| Knowledge gap and confidence | Training gap across staff groups  PARTICIPANT 4: “I don’t think I’ve had any training on vision…at all”  PARTICIPANT 4: “You just don’t and then if I’m like looking after a patient who is like I’ve been told oh they’re partially blind its like well, you’re not giving me any more information what to do with that, like what am I gunna do to help them, cos I don’t know anything  Moderator 1: Uhm yh  PARTICIPANT 2: And that’s the really important thing as well when it comes to assessing vision it then needs to be then well what do I do with this information?”  PARTICIPANT 2: “…a lot of the time people aren’t comfortable with doing certain assessments because ok if you find out there’s a deficit now what?”  PARTICIPANT 2: “I just don’t know if there’s an awareness in healthcare in general about what the difference is and how that feels and then if our patients can’t tell us or say they’ve only come in with their reading glasses and then we put their glasses on and then that’s gunna massively affect their far away vision”  PARTICIPANT 1: “Should there be (a guideline), yes, is there no”  Preferred training modes  PARTICIPANT 2: “…putting them in their shoes” (using simulation specs) | Training gap across staff groups  PARTICIPANT 6: “I think it's probably like as much as our, our team are capable of recording,”  PARTICIPANT 6: “…that'd be useful to know as well. It's like, what else can we offer?”  PARTICIPANT 8: “…they (patients) are getting confused because they might be have the both glasses, they don't know which one is reading glass, which one is the vision glass. So they will get confused also, especially the dementia patients in our wards.”  PARTICIPANT 7: “…there there’s patients wearing another patient's glasses…”  PARTICIPANT 9: “I don't think we've got a guideline in general.”  PARTICIPANT 9: “…when we tell people why we need this vision screening, it's going to help them to accept it.”  Assessing vision in cognitively impaired patients  PARTICIPANT 6: “Especially like lots of cognitive patients. I don't know how I'd possibly try and do like a mini eye test.”  Moderator 1: “What are the barriers to doing that?  PARTICIPANT 6: I think like cognitive deficits are a massive one” | Training gap across staff groups  PARTICIPANT 12: “I've been HCOP, with HCOP for 11 years now. I've never really had specific teachings on eye sight or different ways eyesight can be affected.”  PARTICIPANT 11: “Fundoscopy is all we do. I'm not even great at that to be honest. I always get somebody like. Can you just double check.”  Moderator 1: What was the training like for that?  PARTICIPANT 11: Pants, see one, do one, teach one”  PARTICIPANT 12: “It's also I I don't know much about it, so I don't know if there is any health conditions that can increase problems with vision or anything like that. So it would be interesting to know because we all look at the medical history.”  PARTICIPANT 12: “Yeah, get an understanding ourselves. So we know. Well, I think the more you've got an understanding of things, you know what to do, what to say, what to look at.”  PARTICIPANT 11: “…how many years training is it like 3-4? Five years? (for orthoptics)  Moderator 1:  Yeah, 3 plus.  PARTICIPANT 11:  Three plus. Yeah. So you can't teach somebody what you know in the limited time that you're going to have… recognise any red flags that are, like, really important and things that you don't want us to miss.”  PARTICIPANT 11: “I don't want to say oh I'm competent at assessing someone’s vision. If I then miss something really vital that then impacts the patient’s health.” | Training gap across staff groups  PARTICIPANT 13: “So we do like coordination tests. We'll do like like visual field testing, That's like, that's far. We don't really go that far with physio.”  PARTICIPANT 13: “I guess it's hard for me to say because I've only been here for like 6 months, but so far I've had like, no training on vision at all at university we did have like a module… part of our neurological assessment”  PARTICIPANT 13: “I probably just hand over to someone else, to a doctor or someone for me to know their visions changed is probably enough.”  PARTICIPANT 13: “I think like to be honest like an actual teaching on it I think would be really good for me.”  Eye specialists don’t get training on falls or health care of older people either  PARTICIPANT 14: “Nope. Not that I am aware of, no.”  PARTICIPANT 13: “…for me to know their visions changed is probably enough for my assessment.”  Preferred training modes  PARTICIPANT 14: “Education, teaching for the doctors, letting them know it could be part of their ward induction pack”  PARTICIPANT 13: “…there's some band 5 like rotational specific ones as well… then every single rotational 5 can experience that teaching”  PARTICIPANT 13: “…it's Physio and OT. So yeah. And we've got assistants on there and activity coordinators that are on there as well. There are lots of people. Yeah. So it's really good.”  Assessing vision in cognitively impaired patients  PARTICIPANT 13: “it’s kinda hard to get, now I think about it, probably accurate things from like some people compared to others.”  PARTICIPANT 13: “A lot of patients have like delirium. That's hard. Dementia, which is difficult and stroke patients, and anyone who’s got like a lot of neurological things going on”  PARTICIPANT 14: “…she felt it was a bit discriminatory (to not vision screen cognitively impaired patients)… but I think logistically, it just, it wouldn't work in that (ward) setting.”  PARTICIPANT 14: “…more bulky, cumbersome equipment, but then I suppose also the the referral pathways as well,”  Physical resources  PARTICIPANT 13: “…people who I can actually contact or to signpost my patients to so that when they are being discharged home, like is they’re anyone in their community or things like the leaflets that you said like have then I could give give to my patients, I feel that would be quite good. I think I don't know if there's any, but like, is there anything I can use on a ward like visual aids, I don’t know?” | Training gap across staff groups  PARTICIPANT 16: “I mean there is such a variety, because it depends on people’s background, like from a therapist point of view, some people worked on stroke, but again that is mainly where I was have my understanding of like assessing.”  PARTICIPANT 15: “I never saw it doing like a training for vision that I remember.”  PARTICIPANT 17: “One actually doing the assessment and two confidently knowing OK, so I've noticed some deficit or impairment, but I don't actually know what that relates to. I'll just refer on to someone else who can look into it more, yeah.”  PARTICIPANT 16:”I can’t remember (the content of the proforma)  PARTICIPANT 15: Yeah. It’s got a lot of questions.”  Assessing vision in cognitively impaired patients  PARTICIPANT 19: ”…(patients with dementia) might not have the patience to do it (the vision assessment), they might be easily agitated.”  Physical resources  A formal vision assessment would inform Doctor referrals  PARTICIPANT 17: “…the doctors are going to want some evidence. No, we're not. You can't just turn up on ward round and go. Yeah. Can you go and make a referral and look into this?” |
| Acute pressures | Equipment and space availability  PARTICIPANT 2: “…it sounds stupid to say it and but let’s be brutally honest, one of the things that prevents me from assessing vision is I’m like now I have to go to the RCP website, print out the pdf,”  PARTICIPANT 2: “So having something that actually is accessible and you can grab and is simple.“  Time and staffing burden  PARTICIPANT 2: “...the RCP guidelines is a really long timely eh so sorry time consuming assessment and I’m just not sure any clinician would have the time to do that on every patient”.  PARTICIPANT 4: “We have a lot of other things to focus on,”  PARTICIPANT 1: “…just getting you to do another, another thing in a list of a hundred jobs I’m tryna do,”  PARTICIPANT 2: “…work out how that fits in our very very busy uh working lives and assessments,”  PARTICIPANT 2: “I think it would help raise awareness, I don’t know if it would help change practice necessarily because of the environment that we’re in in the NHS in terms of and I hate saying this but time constraints and staff shortages and things like that.” | Equipment and space availability  PARTICIPANT 8: “…we don't have any equipments nearby.”  Time and staffing burden  PARTICIPANT 9: “Ummm so far we do a falls risk assessment for most of our patients. It's very reasonable because yeah,  Moderator 1: OK, yeah.  PARTICIPANT 9: Following ageing mobility sometimes declines isn’t it. So I don't think it's bad if it's added for them.” | Time and staffing burden  PARTICIPANT 11: “But it’s finding the time to do that (training).”  PARTICIPANT 11: “I would within my role because we have a a monthly teaching day,”  PARTICIPANT 10: “I think we would because we get protected, we get protected for teaching every week.”  PARTICIPANT 11: “OK. Think sometimes it’s about having the time to then do that, as daft as it is. But you know, if you're in a rush sometimes and you've got XYZ things to do, it's easier, isn't it for you to look up at that clock. Tell them the time, it’s like having kids…”  PARTICIPANT 11: “Time, always time (is a barrier). But I think if for me. To do something like that on everybody, maybe. Is creating more of a barrier because it's then harder.”  PARTICIPANT 11: “…so there’s patients that we know were going to go home again, it would be daft. It would be time consuming and probably a waste of the very precious time to do that on all of those patients, if we know that they're definitely going to be leaving again. But maybe those that are staying, maybe you know. Something that has to be done within 24 hours of a patient’s admission or something?”  Costs  PARTICIPANT 11: “…a lot of it, like comes down to cost efficiency as well, isn't it?” | Time and staffing burden  PARTICIPANT 13: “In my opinion, I'm a bit. I'm one of them, where like if I've got loads of patients to see. I've got loads of patients to see, but I'd rather not like tick a box but make sure that I’d rather actually just spend quality time with them and know that what I'm doing is working. So if it means that like I have to add an extra step… that's absolutely fine by me”  PARTICIPANT 14: “…everyone's trying to achieve something different, aren't they? So just physically being able to get to the patient at a time when they’re not with someone else.”  PARTICIPANT 14: “You kind of feel and you have to go hunting for everything, and no one knows who you are necessarily.”  PARTICIPANT 14: “The list (of patients) that I pull off (to vision screen) is getting bigger… I think there's more than there used to be. And whether that’s because people are getting older. I don’t know.”  PARTICIPANT 14: “…if I’m timetabled to go and screen them, yet there are clinical pressures from elsewhere that maybe just more pressing. I might be moved to go and do something else.” | Equipment and space availability  PARTICIPANT 16: “I think a lot of the time relatives like take glasses home as well. Yeah, because they'll be like, oh, you're gonna lose it in hospital.”  Feasibility:  PARTICIPANT 17: “…depends on. What? What? We needed to do. And then the space, the environment that you do it in. So I guess something easy at the bedside that can be replicated anywhere,”  PARTICIPANT 19: “…shouldn’t be anywhere special like, anything like special equipment to do it I think.”  Time and staffing burden  PARTICIPANT 17: “I think if it's indicated, I think a visual assessment should be undertaken, so not just a blanket or all older patients have vision assessments.”  PARTICIPANT 19: “I'm not saying lasts like an hour or something, maybe a couple minutes just to see. And if they want something more in depth…”  PARTICIPANT 15: “Within two hours you have to do this (nursing admission) assessment. Then you focus more on that assessment than others.”  PARTICIPANT 19: “Two hours does seems like a lot of time but it’s not when you consider the amount of things we have to do.” |
| Current practice | Clinical impact of formal assessment  PARTICIPANT 4: “Very basic very basic.”  PARTICIPANT 1: “But again its that tick box isn’t it, actually.”  PARTICIPANT 2: “…if you do an assessment or a care plan every 2 hours it really looses its power in your clinical practice.”  PARTICIPANT 2: ”...do you think that sort of assessment massively changes your practice?  PARTICIPANT 4: No  PARTICIPANT 5: I think its just looking just the presence or absence of different glasses that’s it nothing much more”  PARTICIPANT 4: “Yh it just becomes a ritual every 2 hours,”  PARTICIPANT 4: “…oh its 2 hours again, tick all the boxes, it doesn’t provide anything.”  PARTICIPANT 4: “I think glasses just seem to be like an add on,”  PARTICIPANT 4: “You’re not really using that... or actually know the truth about it,  PARTICIPANT 1: “It’s just very tokenistic isn’t it, ah I’ll ask something about eyes”  PARTICIPANT 4: “If it was like maybe a slightly bigger assessment that you do, I don’t, I don’t really know how often it would need to be done  Moderator 1: Umhm  PARTICIPANT 4: Then it wouldn’t just feel like, like it would actually feel like a useful tool. And then something like we could look back on,”  PARTICIPANT 1: “…it would be best to have it as they come in”  PARTICIPANT 3: “Doesn’t actually feel that relatable to the patient.  PARTICIPANT 1: To the patient’s care.  PARTICIPANT 3: It’s not person centred”  Informal observational assessment  PARTICIPANT 4: “…they think there’s something on their table and mistake something on their table for something else and then they’re desperately trying to stand up and get at it,”  PARTICIPANT 4: “Depth perceptions not there and then,”  PARTICIPANT 2: “They walk into doors a lot as well, do you find that when you’re like taking people to the toilet, their frame will like catch the door frame or the end of the bed  PARTICIPANT 4: Yh. Cos like spatial awareness seems to be well a problem…” | Clinical impact of formal assessment  PARTICIPANT 7: “…it's not really in depth.”  PARTICIPANT 8: “It's not giving any importance.”  PARTICIPANT 7: “So it's just saying to you it's got glasses. Yeah, it's not saying this patient needs glasses.”  PARTICIPANT 7: “…there should be some some sort of vision assessment done with the admission.”  PARTICIPANT 6: “And it's pretty much the box from what I can remember that says any visual deficits. And I think it's like you might put these glasses or none noted or yes, I guess. And yeah, we should be completing that based on what the patient tells us,”  PARTICIPANT 8: “…mentioning in the nervecentre or their doctors, they are mentioning in the notes…”  PARTICIPANT 9: “They do tell us, except when we when maybe when they are confused, then we fall back on their family to help us in that situation.”  Informal observational assessment  PARTICIPANT 6: “often you're walking with a patient and then their frames will sound like knocking into their bed or they're just like, not having good obstacle clearance or you coming. They're just asking like, oh, it's the toilet nearby and they're looking in front of them and like, really, they should be able to see that there's nothing, nothing there.”  Moderator 2: “Are there any other ways that you observe or see visual problems when you're sort of supporting people in your ward area rather than just mobility?  PARTICIPANT 7: Eat, eating and drinking?” | Clinical impact of formal assessment  PARTICIPANT 11: “…part of that could be asking about vision. There could definitely be something within there as to why they may have fallen. But then would that be another box for somebody to miss. I don’t know.”  PARTICIPANT 10: “…are those glasses working for you? They don't realise until you ask the question. Actually, no, they're not working for me. Maybe I should have got my eyes tested.”  PARTICIPANT 12: “I think also as well, we know we can find out if it's in the proformas like if the visions OK, do they wear glasses but its when did they last get their eyes checked, especially with older people, because their eyesight gets worse over time. So when was the last time they actually had? And we we haven't got anything in there of that.”  Informal observational assessment  PARTICIPANT 10: “I kind of notice it (vision impairment) more when I say can you walk to that chair over there or can you walk the set distance to something visual and then they say where is that? And I'm pointing and they can't see. Or if a patient's bumping into things, then I'll start to question it.”  Moderator 1: “…how do you know if the patient has really bad vision?  PARTICIPANT 11 1:  Asking them.”  “past medical history”  PARTICIPANT 12: “But it's it's just one of those things that you don't actually think about. So thinking about it. We actually do it on the every day, quite regularly. You don't realise it's, I don't know.” | Clinical impact of formal assessment  PARTICIPANT 14: “The level of history that I'm given by the patient can sometimes make it difficult to accurately interpret the results that I've got.”  PARTICIPANT 13: “So right on my initial assessment of someone, I’ll know if they wear glasses or if they don't, then I know if I'm looking out for something as well, I guess.”  Informal observational assessment  PARTICIPANT 13: “So a lot of them will walk with, like walking aids, are they like over shooting to hold on to it. Are they a bit wobbly,”  PARTICIPANT 13: “I would say 90% what I observe and then probably 10% of, I will check people's vision”  PARTICIPANT 14: “Sometimes, like you know, like if you follow someone with your eyes and they can't follow me or it sometimes it seem as if they're distracted”  PARTICIPANT 14: “…anyone who, you know, the big one is like startled when I start talking to them”  PARTICIPANT 13: “…it seems as though they're going to sleep, or they just don't want to engage but it’s actually that they've got double vision and they like can’t bare to look at me.” | Clinical impact of formal assessment  PARTICIPANT 16: “I think it, it literally just says any issues reported with the visions. It's quite a broad question is, it does actually say? I don’t know…”  PARTICIPANT 17: “I think having a quick assessment of the vision during the maybe initial assessment would be good.”  PARTICIPANT 16: “There's like a admission booklet at the beginning of the note, so I guess the first thing would be you look at them and they have a list of medical history and also speaking to the patients, you know like visually you might see glasses near them or they might, they might tell you or you can ask. Family members…”  PARTICIPANT 19: “So the HCA or the Nurse hands over to me this person is visually impaired, it might even say on their board not to give it to the patient, or to make sure that others are aware.”  PARTICIPANT 19: “I think a formal assessment would be useful. Yeah because sometimes I might be with the patient for one shift, not knowing anything about the vision and next shift someone might hand over to me saying oh by the way, she can't see,”  Informal observational assessment  PARTICIPANT 17: “…their eye contact, are they looking at us? Are they able to recognise objects if we're asking them to use objects, pick up objects, if they're missing things when they’re going to reach for things,”  PARTICIPANT 15: “Can you give me your hand? And after that they say I can’t see proper or I can’t see that side. Can you come in that way because this eye is blank.” |
| Roles and responsibilities | Role of falls MDT  PARTICIPANT 2: “I would love to one day have a multidisciplinary CGA-based falls clinic at … with a physio, and orthoptist, geriatrician, specialist nurse, OT,”  PARTICIPANT 1: “Should it be part of a CGA, so it’s part of something that the medical team are doing would it be kind of the nursing staff, is it therapies, is it a shared responsibility? Whoever gets there first? Should it be being done at the point of coming to ED?”  PARTICIPANT 2: “…therapies are probably best placed to introduce visual screening but that only works if you’re on a ward where you’re seeing every patient and then you have to think about weekends.”  Patient knowledge and engagement in self-care  Asking patients to go to the optician on discharge:  PARTICIPANT 1: “…sometimes it feels like it’s an empty (gesture)”  PARTICIPANT 4: “I’ve had patients before, given them their glasses and they’re like well they don’t really do anything and I’m like well why not and they’re like oh they’re not the right prescription.  PARTICIPANT 1: These are me old ones that I’ve accidentally grabbed  PARTICIPANT 4: Or yh or they’re like oh I’ve not been I’ve not been and got them my eyes tested for a while and I’m like oh, alright then.” | Role of falls MDT  Moderator 2: “Whose responsibility do you think it is to do that assessment and to come up with a plan?  PARTICIPANT 7: It's everybody's really.”  PARTICIPANT 9: “Yeah, this is really nursing and therapy. Yeah. So if we are concerned, then we can involve the doctors.”  PARTICIPANT 6: “I think if anything it could be more, I'd imagine it'd end up being more like a therapy thing just because like in theory we're the ones saying how someone should be mobilising. And if we think that they're not safe to mobilise a certain way because of vision, maybe that should be on us to like assess and to hand that over to like nurses and HCAS.”  PARTICIPANT 7: “…as healthcare assistants you're the first ones really because you guys might not get round to that patient until because you have a list don't you go round to.”  PARTICIPANT 7: “…you tend to see a lot more of what they're doing and what they can't do (as HCA’s).  Patient knowledge and engagement in self-care  PARTICIPANT 7: “…they don't remind themselves that they need glasses…and sometimes glasses are hid in cupboards or they're not out to say it there's nothing to say this this patient needs glasses or so do you know what I mean?”  PARTICIPANT 8: “…they (patients) are used to with the things because they are not newly diagnosed with their eye or vision problems. So they are even, they are also trying their level best to don't disturb others….” | Role of falls MDT  PARTICIPANT 11: “…I personally I need to also take my own training into my hands a bit more…”  PARTICIPANT 11: “Every part of this (clerking proforma) should be filled in and some people do better than others at filling it in.”  PARTICIPANT 10: “To some degree, we all should be responsible because if there’s a problem it needs to be dealt with.”  PARTICIPANT 11: “…whose responsibility is it to do that? Is that like a physio role? Is it a nursing role? Is it medical? Remembering that our junior and our residents. Sorry, not junior Drs anymore, they rotate every four months.”  Patient knowledge and engagement in self-care  PARTICIPANT 11: “Probably benefits certain patients that would have difficulty accessing opticians.”  PARTICIPANT 12: “I think also when they're in their own environment they probably don't feel like it's as important either because it's a familiar environment… they have to rely on the eyesight a little bit more. Yeah, so maybe it's not a priority cause at home, you know, they know where everything is.”  PARTICIPANT 11: “Maybe they (patients) feel like they're not going to die?... getting to my appointment about my heart is more important than getting to my appointment about my eyes.”  PARTICIPANT 11: “If it's (the vision), you know, gradually changed over time, maybe they don't. You know, that's just their normal, isn't it? They don't realise until then, they put on some glasses that are the correct prescription. They're like, oh, this is what the world is meant to look like.”  PARTICIPANT 12: “But if we just send them home, how do we know that they're going to have that check up? Are they going to come in again with a fall because they have got issues with their eye sight.  PARTICIPANT 10: But then is that not their (the patient’s) responsibility to do that? Or the family, or the carers, to sort that out, as opposed to us?”  PARTICIPANT 11: “I think and not to sound heartless, but the patient also has a responsibility to themselves... you can lead a horse to water you can’t make it drink.”  PARTICIPANT 11: “…you know there's a lot of barriers aren’t there… the patients got dementia. So they're not going to know so then you rely on a relative. To take the, you know, relying on how mobile the patient is… sometimes maybe it's not something that’s prioritised with their health” | Patient knowledge and engagement in self-care  PARTICIPANT 14: “Personally, I think testing them (patients) in hospital while they're having everything else done is beneficial…could take a really long time before they feel comfortable and are brave enough to go out to the optician“  PARTICIPANT 14: “I think that with the elderly, they often make the assumption that if their vision is changing, nobody will do anything about it, so they're more likely to ignore it than a younger person,”  PARTICIPANT 14: “They can see everything in their homes that they want to see. So as far as they're concerned…”  PARTICIPANT 14: “I can’t be bothered with another appointment, not another appointment”  PARTICIPANT 14: “And they don't necessarily realise how bad their vision is because they're in their little bubble.”  PARTICIPANT 14: “It's all what the patient views is important and sometimes it's the fact that their eye history is 30 years ago, so. And they don't always think to tell you.” | Role of falls MDT  PARTICIPANT 17: “We'll ask the nurses.”  PARTICIPANT 17: “We can contribute a picture towards that as therapists can’t we? …We checked with the nurses. The HCA.”  PARTICIPANT 19: “I think everyone should be able to, a nurse HCA physio, should all be able to do it just because we have different, we look after the patient from different kind of aspects… We do get busy on the ward. We might not be able to do all of the visual assessments. I might be able to do 1 or 2 and then the nurse could do 2.”  PARTICIPANT 17: “…depends on the assessment. If it's something that anyone can do with a bit of training, yeah, then yeah, we should all do it.”  Patient knowledge and engagement in self-care  PARTICIPANT 17: “…you know when patients come into hospital and they we treat them like patients and they feel like patients. And so it's like all their personal belongings are taken away and I think glasses is also… And sometimes they don't tell us, so we have to look at the visual cues…”  PARTICIPANT 17: “You know what I'm thinking though, if if I picture my patients in my mind’s eye, that actually, there's not many that you approach that actually wear glasses all the time.” |
| Key: FG- Focus Group, P-Physiotherapist, HCA- Health Care Assistant, N-Nurse, OT-Occupational Therapist, OR- Orthoptist | | | | | |
